# A predictive analysis on the risk of peste des petits ruminants in livestock in the Trans-Himalayan region and validation of its transboundary transmission paths

**DOI:** 10.1371/journal.pone.0257094

**Published:** 2021-09-10

**Authors:** Zan Zeng, Shan Gao, Hao-Ning Wang, Li-Ya Huang, Xiao-Long Wang

**Affiliations:** 1 College of Wildlife & Protected Area, Northeast Forestry University, Ministry of Education, Harbin, Heilongjiang Province, The People’s Republic of China; 2 Key Laboratory of Wildlife Diseases and Biosecurity Management of Heilongjiang Province, Harbin, Heilongjiang Province, The People’s Republic of China; 3 School of Geography and Tourism, Harbin University, Harbin, Heilongjiang Province, The People’s Republic of China; 4 Changbai Mountain Academy of Sciences, Antu, Jilin Province, The People’s Republic of China; University of Lincoln, UNITED KINGDOM

## Abstract

Although the Trans-Himalayan region (THR) is an important endemic and rendezvous area of peste des petits ruminants (PPR), monitoring and prevention measurements are difficult to execute because of the rough geographical conditions. Besides, a heterogeneous breeding system and the poor veterinary service of susceptible animals compound the existing problems. Here, we propose a forecasting system to define the key points of PPR prevention and aid the countries in saving time, labor, and products to achieve the goal of the global eradication project of PPR. The spatial distribution of PPR was predicted in the THR for the first time using a niche model that was constructed with a combination of eco-geographical, anthropoid, meteorological, and host variables. The transboundary least-cost paths (LCPs) of small ruminants in the THR were also calculated. Our results reveal that the low-elevation area of the THR had a higher PPR risk and was mainly dominated by human variables. The high-elevation area had lower risk and was mainly dominated by natural variables. Eight LCPs representing corridors among India, Nepal, Bhutan, Bangladesh, and China were obtained. This confirmed the potential risk of transboundary communication by relying on PPR contamination on the grasslands for the first time. The predicted potential risk communication between the two livestock systems and landscapes (high and low elevation) might play a role in driving PPR transboundary transmission.

## Introduction

Peste des petits ruminants (PPR) is a contagious viral disease that primarily affects domestic and wild small ruminants. Because of its high morbidity and mortality, PPR is responsible for heavy economic losses in livestock husbandry across many developing countries. It is considered a significant threat to the global goat and sheep industry [1]. PPR is caused by peste des petits ruminants virus (PPRV), a member of the family *Paramyxoviridae* and genus *Morbillivirus* along with Distemper, Rinderpest, and Measles viruses [2]. To date, four PPRV lineages (I-IV) have been identified worldwide. Lineage IV is the dominant strain in Asia, including the entire Trans-Himalayan region (THR) [3]. PPRV has a tropism for epithelial and lymphoid cells [4]. The virus can exist in different host body tissues and is discharged from the body through various secretions and excretions. These secretions and excretions, including the respiratory droplets, become the source of PPRV, allowing the transmission of the virus through close contact [5] and aerosols [6]. The clinical symptoms of PPR typically begin with dullness and fever (>40 °C). Subsequently, there is the development of oral mucopurulent discharge, ocular discharge, and eventually, oral lesions, bronchopneumonia, and diarrhea [4]. The severity of this disease is determined by the strain of the virus, local environmental features, and the immune status of the infected host [7]. The morbidity and mortality of PPR can vary between 10%–90% and 50–90%, respectively [8]. The typical latency period of PPR is 4–6 days, whereas the longest incubation period reported is 21 days [9]. The major hosts of PPRV are livestock, such as sheep and goats [10]. Wildlife is also an important target for PPRV. The main targets are antelope (such as saiga antelope—*Saiga tatarica mongolica*), ibex (such as Siberian ibex- *Capra sibirica*, Sindh ibex—*Capra aegagrus blythi*), gazelle (such as goitered gazelle—*Gazella subgutturosa*, Arabian gazelle—*Gazella arabica*) [11–17]. In Asia, clinical signs and mortality of PPR in wildlife have been reported essentially as the same as those in livestock [14–19], which provides a basis for interspecific transmission. This situation is different from the non-clinical infection in Africa, i.e. no viral shedding even if an antibody is produced [20]. Especially in THR, bharal (*Pseudois nayaur*) [18], markhor (*Capra falconeri*) [13], blackbuck (*Antilope cervicapra*) [21], and Himalayan goral (*Naemorhedus goral*) [22] have been found to exhibit obvious clinical signs. Among them, strains from bharal and markhor were successfully isolated. Phylogenetic analysis showed that they were closely related to the strains isolated from livestock, suggesting a potential relationship between them [13, 18].

PPR was first reported in West Africa in 1942 [23] and spread across Africa and Asia. Available research indicates that China, India, and Nepal are all PPR epidemic countries. Two PPR epidemics have been documented in China; the first occurred in 2007 in the Tibet Autonomous Region of China [24]. The more severe outbreak occurred between the end of 2013 and the first half of 2014, which was first identified in the Xinjiang Uygur Autonomous Region and later spread to more than 20 provinces in total. This consequence in more than 30,000 sheep infections, of which 10,000 animals died [24].

In response to the heavy losses caused by PPR, the World Organization for Animal Health (OIE) and the Food and Agriculture Organization (FAO) have set the goal of eradicating PPR globally by 2030 [25]. It has been suggested that the global eradication of PPR could return benefits of about $74 billion over 15 years [26]. However, the continuous epidemiological cycles of PPR worldwide [27] constitute a great challenge to eradicating the disease. This calls for a deeper understanding of its temporospatial characteristics. The THR is an important endemic and high-risk area of PPR where monitoring and prevention measurements are difficult to implement. This can be majorly attributed to the rugged natural geographical conditions and the low effective livestock system. The poor veterinary services further complicate the situation in the region. Thus, a forecasting system would be a strong aid in defining the key points of prevention to save time, labor, and products for underdeveloped countries and regions.

According to reports, the PPR risk exists across the THR, in which livestock serve as the maintenance hosts [28]. Wildlife possibly plays the role of bridge hosts [29], in which virus transmission is not maintained but can persist for a while and be transmitted back (spillback) to livestock [30]. Although scientists argue for the direct epidemiological linkage at the interface of livestock and wildlife [31], the interspecies transmission of PPRV during grazing and at water sources has been confirmed [32]. Sharing the use of rangelands by livestock and wildlife can lead to disease transmission [33]. Abubakar et al. [14] pointed out that an outbreak of PPR in Sindh ibex was due to the spillover of the virus from a recent outbreak of PPR in nearby domestic small ruminants. Similar PPR spillovers to wild hosts are reported in Tibet [18] and the Ngorongoro Conservation Area in northern Tanzania [20]. Except for trade, free migration of wildlife and nomadism-driven back and forth movement of livestock enable their meeting in the same space (contaminated or not) across time. Both animals and humans prefer low-energy-consuming surfaces during movement, which has become its driving force [34]. If the migration and grassland sharing of the multiple PPRV hosts last, reliance on the contamination of habitats within the latent period of the disease is expected, and the probability of direct contact via contaminated grassland is increased. While it would be arbitrary to conclude that the infection occurred on the cross-country paths, the potential communication of risk among the Trans-Himalayan neighboring countries is worth monitoring.

We assume that interspecies transmission of PPRV occurs on small ruminants in THR, which forms the basis for the transboundary transmission of PPR. Initially, we predict the distribution of PPR on both sides using the maximum entropy model (MaxEnt) and the connectivity of landscapes among different PPR-contaminated regions using the LCP model, thereby revealing the potential transboundary communication of PPR.

## Materials and methods

### Research area

**Fig 1:**
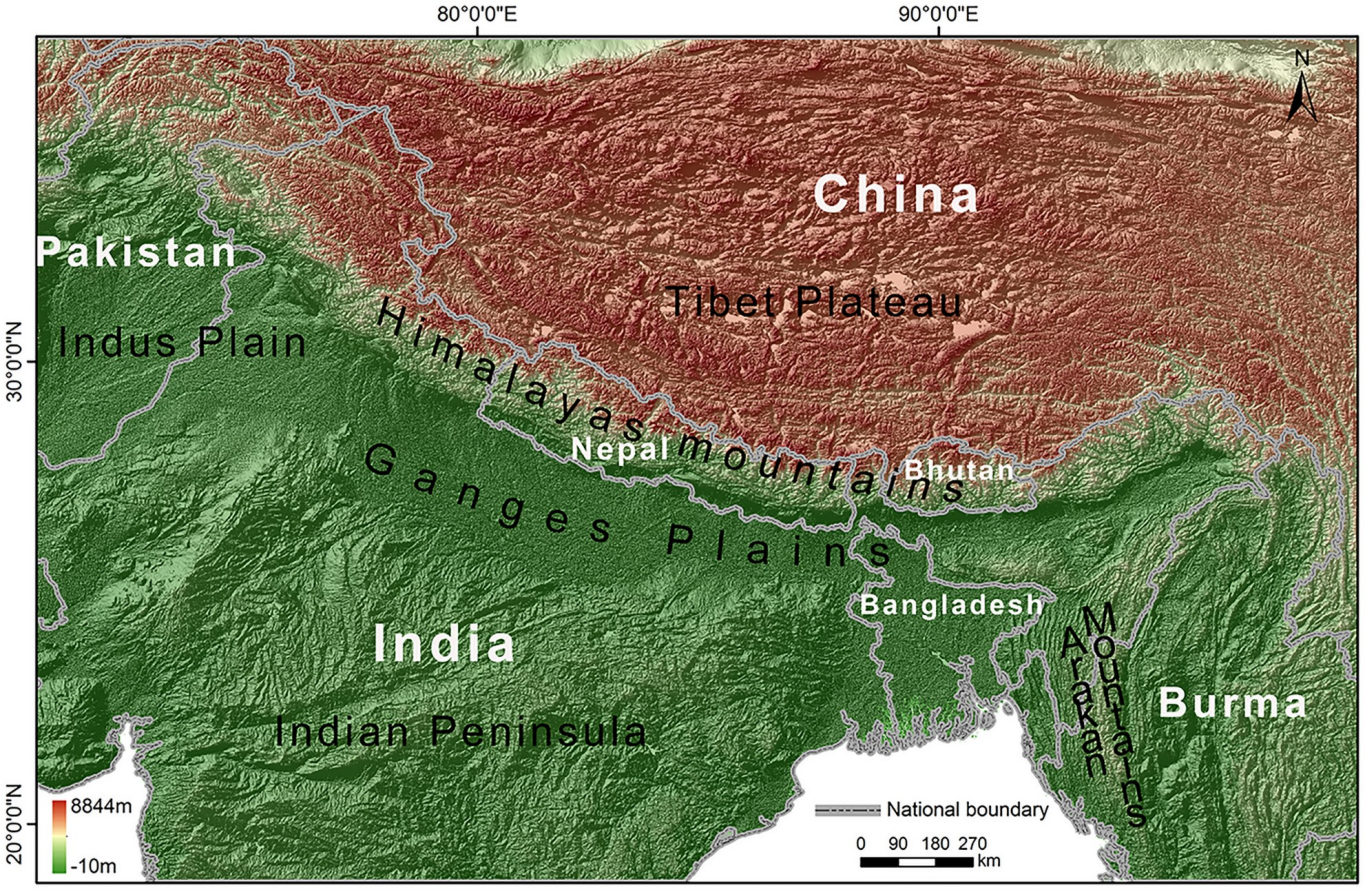
Location map of THR. The elevation is depicted by the digital elevation model (DEM). DEM was obtained from USGS Earth Explorer (https://earthexplorer.usgs.gov); the boundary was obtained from Natural Earth (http://www.naturalearthdata.com/), which is a schematic line illustrating the relative position of each country and should not be re-used or misinterpreted for any political reason.

Our research area (Fig 1) is defined as the THR, which mainly included the Himalayan mountains, a part of the Tibet plateau, the Ganges plains, a part of the Indus plains, a part of the Indian Peninsula, and the Arakan Mountains. Altogether, seven countries are in this region, including China, India, Nepal, Pakistan, Bhutan, Bangladesh, and Burma, covering approximately 6.89×10^6^ km^2^. The northern, central, and southern parts of the THR differ in natural geography, ecology, and climatology. The central part, i.e., the Himalayan mountains, has the highest elevation of approximately 4000–8800 m. It is a long and narrow mountain range with a length of approximately 3000 km and a maximum width of only 400 km, covering an area of more than 1×10^6^ km^2^ [35]. In the south-facing slope of the mountain, lower elevation regions were covered by the evergreen broad-leaved forest, and higher elevation regions were covered by coniferous forests, shrubs, and alpine meadows. The north-facing slope displayed the alpine climate, dry and cold with little precipitation [36]. The northern and the southern parts are divided by the Himalayan mountains. The northern part is constituted by the Tibetan Plateau, with an elevation of approximately 2500–5000 m. This area is dominated by plateaus and mountains interspersed with plains and basins. The intense radiation, low temperature, large daily temperature range, and small annual temperature range verify a typical plateau climate feature [36]. The elevation in the southern part was below 1500 m. It mainly consists of flat, fertile plains with a tropical monsoon climate and a subtropical grassland climate [37].

There are two different livestock systems in THR. The mixed crop-livestock farming system is represented by the Ganges plains, the Indus plain, and the Indian peninsula (low-elevation). The other one consists of the grazing system, represented by the Tibetan Plateau (high-elevation) [38]. The Tibetan Plateau is a traditional pasture (the number of sheep ≈ 10 million, goats ≈ 5 million) [39]. India (sheep ≈ 65 million, goat ≈ 135 million), Pakistan (sheep ≈ 3.7 million, goat ≈ 7.4 million), and Nepal (sheep ≈ 0.8 million, goat ≈ 10.9 million) are also the core areas of animal husbandry in the world [40]. With respect to free susceptible hosts, bharal is distributed in the Tibetan frontier, Nepal, and Bhutan, and population densities in Nepal were found to be 0.9–2.7 individual/km^2^, increasing to a maximum of 10 in the winter, as herds congregate in the valleys [41]. The number of mature individuals is approximately 47,000–414,000 [42]. Himalayan goral occupies the south-facing slope of the Himalayan mountains, and its population density varies from 2.6–10.5 individuals/km^2^ [43]. Blackbuck is widely distributed in the Indian subcontinent, and the number of mature individuals is around 35,000 [44]. Markhor is mainly distributed in Pakistan. It is also found in small numbers in India (Jammu Kashmir). The number of mature individuals of this species is approximately 5,754 [45].

### Research data

There were 1135 recorded PPR outbreak locations collected from the OIE reports and published studies [24, 46–51], including 107 records collected from the latter. The host datasets were used alongside four fundamental environmental predictor categories relevant for habitat modeling of terrestrial macro-fauna, i.e., climate, terrain, vegetation, and human impact [52] (Table 1) to construct the environmental model in this study. The preprocessing and calculation of all spatial data were conducted in ArcGIS 10.6 and projected in UTM-WGS-1984 with standard settings or resampling to 30 arc-seconds.

**Table 1:** Data layer and source, raster/vector, value range/categories (number of subcategories in brackets), and specification of the unit of measurement/impact (proxy).

Layer	Source	Value/categories	Variable/proxy
**Climate** ^a^			
Monthly P	CHELSA	0 to 275 mm/month	Precipitation
Monthly mean T	CHELSA	-32.6 to 37.3°C	Mean Temperature
Monthly min T	CHELSA	-37.3 to 30.5°C	Minimum Temperature
Monthly max T	CHELSA	-27.9 to 43.6°C	Maximum Temperature
Bioclimatic	CHELSA		Annual trends, seasonality, extreme or limiting environmental variables
ISR-spring	ASTER-GDEM	8.1 to 84.2 wh/m^2^	Topo-climate
ISR-summer	ASTER-GDEM	12.1 to 97.3 wh/m^2^	Topo-climate
ISR-autumn	ASTER-GDEM	3.1 to 60 wh/m^2^	Topo-climate
ISR-winter	ASTER-GDEM	2.8 to 92.2 wh/m^2^	Topo-climate
**Terrain**			
Elevation	ASTER-GDEM	-10 to 8844 m a.s.l	Climbing distance
Slope angle	ASTER-GDEM	0 to 88.2°	Climbing effort
Distance to river	ASTER-GDEM	0 to 410.7 km	Water source
**Human impact**			
Human population	WorldPop	0 to 14229 persons/km^2^	Human-Animal interaction
**Vegetation** ^b^			
Land cover	ESA	Cropland (3), Herbaceous, Tree (9), Shrubland (3), Grassland, Urban areas, Bare areas (2), Mosaic shrub & herbaceous cover, Water bodies, Permanent snow, and ice	Animal food and refuge
**Host** ^c^			
Sheep density	GLW3	0 to 447.5 individual/km^2^	Host-disease interaction
Goat density	GLW3	0 to 1865.1 individual/km^2^	Host-disease interaction

^a^T = temperature; P = precipitation; Source: CHELSA 1.2 (http://chelsa-climate.org/) at 30 arc-second resolution; ISR = Incoming Solar Radiation.

^b^Source: Land cover map (https://maps.elie.ucl.ac.be/CCI/viewer/); the number of subcategories in parentheses.

^c^Source: Gridded Livestock of the World (https://livestockdata.org/contributor/gridded-livestock-world-glw3).

### PPR spatial distribution model

The MaxEnt model is regarded as one of the best-performing specialty distribution modeling techniques for analyzing presence-only data [53]. It creates ecological niche models by combining presence-only data with environmental variables using a machine-learning approach known as maximum entropy. The reliability of MaxEnt has been confirmed by its good capacity to predict novel presence localities for poorly known species/diseases [54]. It has been widely used in many diseases, including PPR [55] and African swine fever [56].

The MaxEnt model is applied to the spatial distribution model building to explore the risk situation of PPR in the THR. In the construction of the model, the regions with significant differences in elevation are treated separately to overcome the problem that the model is not robust enough to deal with the DEM with large differences [57]. The low-elevation model (Model 1) and a high-elevation model (Model 2) were constructed for regions below and above 1500 m, respectively, according to the elevation standard of highland climate [58]. The spatial autocorrelation was minimized by filtering all recorded PPR locations using the SDM Toolbox v1.1c in ArcGIS 10.6 [56]. Filtering was performed by limiting the minimum distance between each pair of points. In addition, the filtering program plays the role of systematic sampling. It can delete adjacent records to reduce spatial aggregation, which is regarded as the most effective method in correcting sampling bias [59]. Multicollinearity was reduced for both the climate and non-climate predictors. First, major predictors were selected using principal component analysis (PCA). The variables with eigenvalues larger than 1.0 and the scree plot criterion or ‘broken stick’ stopping rule for PCA in item-level factoring were adopted [60]. Suppression of unnecessary loading and rotation of factor pattern of variables was used to retain predictors for subsequent analysis in MaxEnt [61]. Next, variables with low contribution rates were filtered out using the MaxEnt model [62]. Finally, variance inflation factor (VIF) analysis was conducted to evaluate the multicollinearity among predictors after the reduction. A VIF value below 10 indicates low and acceptable multicollinearity [63]. The filtered PPR locations and predictors were then used as input data to construct the PPR model using the MaxEnt algorithm. The present models were developed using occurrence data and 10,000 random background points, representing the distribution of environmental conditions in the study region [64]. We divided the selected presence records into 70% training and 30% testing portions to build and validate the models based on 10 bootstrap replicates. For the remaining parameters, we kept the default settings in the pilot study. Predicted PPR risk maps obtained by models 1 and 2 were overlaid using the fuzzy overlay to construct the final PPR risk map of the THR. For visualization, the Jenks natural break optimization method was used to classify the model output to identify high-risk areas [65]. Smoothing was followed for map visualization [62].

The key component of the model validation procedures is the criterion that evaluates the model performance. We use threshold-dependent and threshold-independent criteria. The area under the ROC curve (AUC) is a threshold-independent criterion based on plotting the true positives against the false-positive fractions for a range of thresholds in prediction probability. Currently, the AUC is considered as the best criterion for assessing model success for presence/absence data [66]. As a threshold-dependent validation measure, we used confusion matrix-based measures, including the Kappa test [67] and correctly classified instances (CCI) [68]. The Kappa statistic normalizes the overall accuracy by the accuracy that might have occurred by chance alone. The percentage of CCI was defined as the rate of correctly classified cells. The thresholds of these two criteria are determined using the sensitivity-specificity sum maximization approach [69].

### LCP model

The least-cost paths (LCPs), the shortest paths between two points with maximum efficiency for a moving individual, have been advocated as an effective, operational, and flexible approach to analyzing connectivity in heterogeneous landscapes [34]. The LCP model allows the integration of multi-dimensional information, including geographic and behavioral information, to comprehensively predict the potential transboundary (transregional) path of the animals. LCPs are employed mainly to determine sites that are potentially used as dispersal routes for terrestrial animals and have been proven to be applicable in ruminants [70]. To predict the potential transmission paths of the PPR in the research area, we created a cost/resistance surface for the migration of small ruminants using land cover type and elevation as cost variables according to their movement capability. Two variables were reclassified using the Jenks natural break method [71]. Cost measurement scale of 1 (lowest cost) to 9 (highest cost) is determined according to the number of the land cover type (except for water bodies, which has been assigned as “restricted’ due to its relative barriers to ruminants) [72]. The goat still maintains the same hoof structure as the wild goat, which is designed for movement and grip in rugged environments [73]. Similar climbing skills, food and shelter requirements make domestic and wild goats have similar movement capabilities and habitat preferences [74]. Cost values were assigned to each classification based on small ruminant habitat preferences (see S1 and S2 Tables for more details) [75–77]. Land cover and elevation were then combined using a logical overlay operation [70]. Recorded PPR outbreak locations were then clustered by K-nearest neighbor cluster analysis, and LCPs between the clusters were analyzed using the constructed cost surface. After the calculation, transboundary paths were highlighted by removing the internal paths.

A sensitivity analysis was performed to assess the robustness of the outputs when they were affected by uncertainty. The main source of uncertainty in evaluating potential paths for host movements is the cost value used to constitute the cost surface. The one-at-a-time method was employed in the sensitivity analysis. This common approach involves changing the input criteria one at a time to observe the effect it produces on the output [78]. This process was repeated for each variable [79]. We changed the cost of the different land cover types/elevation classifications one at a time by adding or subtracting an amount Δ = 5% or Δ = 10% from the original cost value [80] to build iterative models. Raster datasets presenting cost surfaces were produced through every iteration. To measure the outputs, we rely on the Spearman rank correlation coefficient [81] to compare the ranking of countries obtained with original cost values with those obtained with different land cover/elevation cost values. The closer the Spearman rank correlation coefficient is to 1, the more similar the iteration is to the original model. That is, this classification has less impact on the model.

## Results

### Results of PPR spatial distribution models

**Fig 2:**
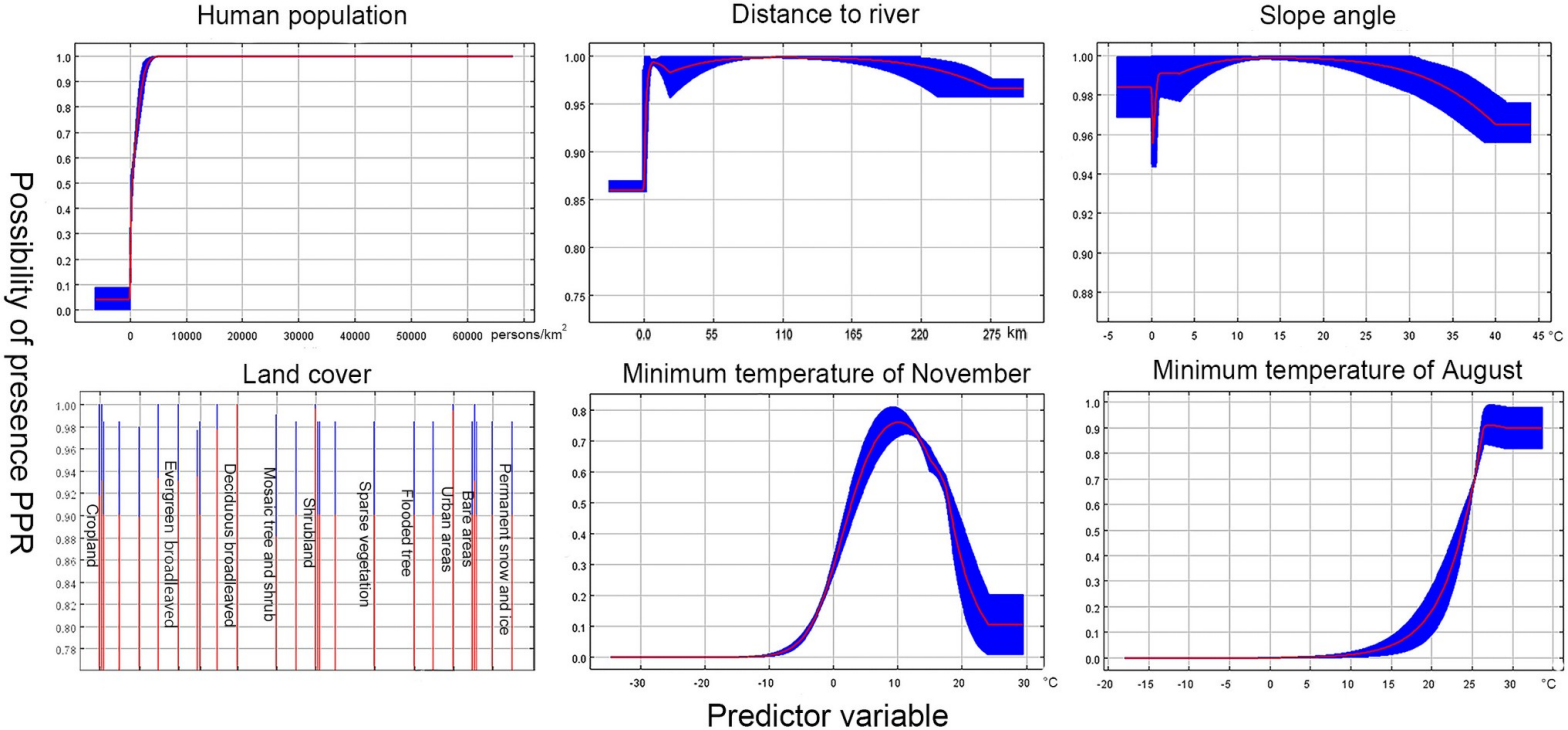
The response curves of model 1 (≤1500 m).

Model 1 (≤1500 m): Altogether, 129 recorded PPR outbreak points at a distance of at least 10 km away from each other were obtained after filtering. After PCA and MaxEnt filtering, minimum temperatures of August (Min T Aug.), minimum temperatures of November (Min T Nov.), human population, land cover, distance to the river, and slope angle were left. The VIF values among the remaining predictors were 1.014–1.742, which was in line with the low multicollinearity standard (<10). Moreover, AUC = 0.892, SD (standard deviation) = 0.002, Kappa = 0.869, and CCI = 0.869, indicated the robustness of the model. The response curves of the different predictors are shown in Fig 2, and the relative contributions of each predictor are shown in Table 2 (left).

**Table 2:** Estimates of relative contributions of the predictor variables to model 1 (left) and model 2 (right).

Model 1 (≤1500 m)			Model 2 (>1500 m)		
Variable	Contribution %	Permutation importance	Variable	Contribution %	Permutation importance
Human population	58.2	36.6	Mean T Apr.	50.8	70.9
Land cover	28.6	10	Land cover	21	5.7
Min T Nov.	5.4	13.7	Human population	17.1	17.1
Min T Aug.	5.2	37.6	Distance to river	8.9	4.4
Distance to river	1.8	1.2	Slope angle	2.2	1.9
Slope angle	0.8	1			

**Fig 3:**
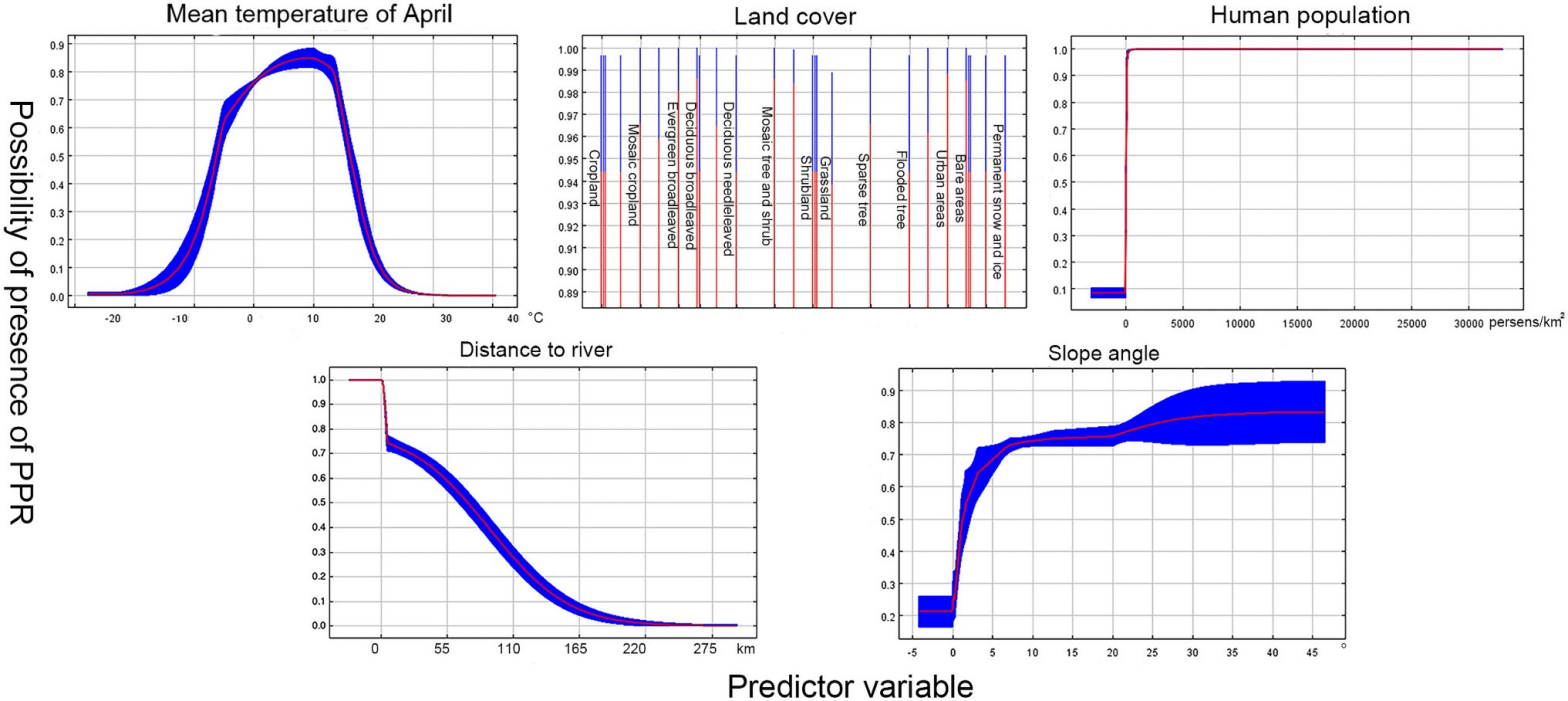
The response curves of model 2 (>1500 m).

Model 2 (>1500 m): A total of 96 recorded PPR outbreak points remained after filtering for 5 km. After PCA and MaxEnt filtering, the mean temperature of April (Mean T Apr.), human population, land cover, distance to the river, and slope angle predictors were left. The robust VIF values among the remaining predictors were 1.006–1.062. For validation of the model, AUC = 0.934, SD = 0.010, Kappa = 0.880, and CCI = 0.881, indicated the robustness of the model. The response curves of the predictors are shown in Fig 3, and the relative contributions of each predictor are shown in Table 2 (right).

**Fig 4:**
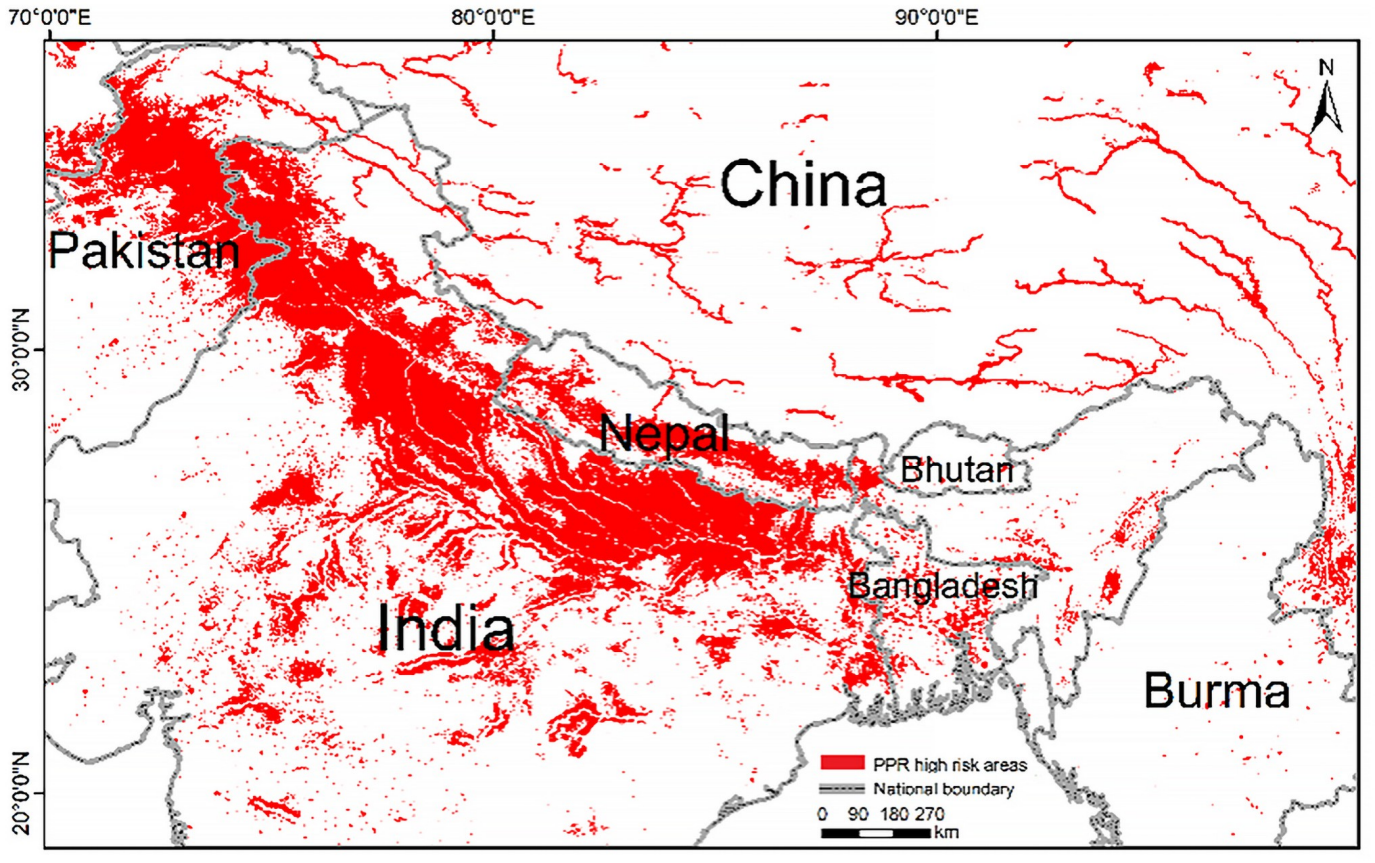
PPR high-risk areas predicted by the MaxEnt model. This map was made in ArcGIS 10.6 using the resulting rasters produced by MaxEnt. The boundary was obtained from Natural Earth (http://www.naturalearthdata.com/), a schematic line illustrating the relative position of each country and should not be re-used or misinterpreted for any political reason.

PPR high-risk areas in the THR were predicted using both models and are shown in Fig 4. High-risk areas were distributed along the Himalayas, covering northern India, Nepal, and central Pakistan. In addition, PPR high-risk areas were scattered throughout Bangladesh and central India. It is worth noting that in Tibet, China, high-risk areas show an obvious trend of distribution along rivers. The results show that the risk of PPR around Pakistan, India, Nepal, and China borders is extremely high. The possibility of transboundary spread cannot be ignored, especially since it may be facilitated by wildlife.

### Results of the LCP model

**Fig 5:**
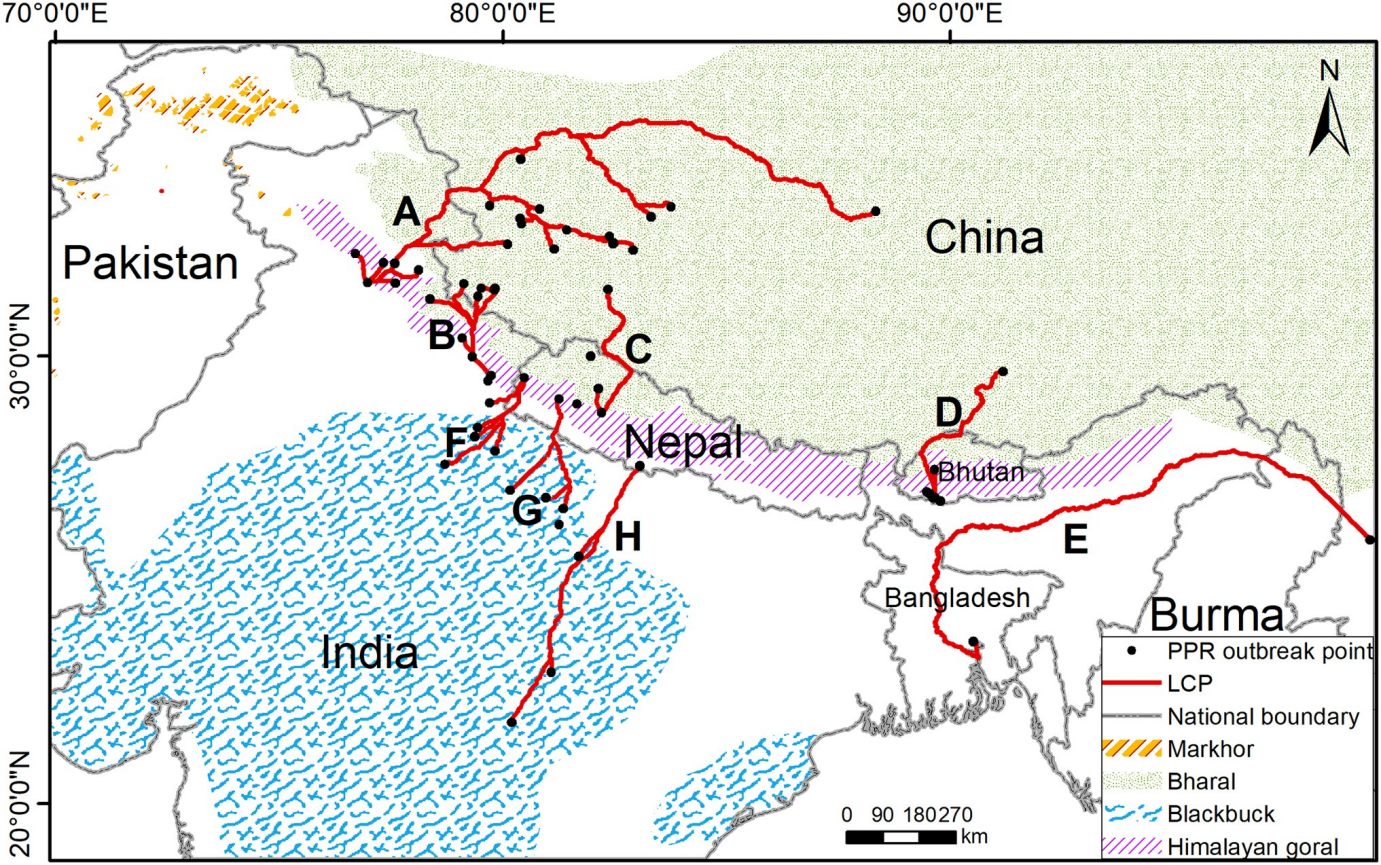
Transboundary LCPs for small ruminants and the distribution of wild ruminants. The territory range of wild small ruminants was obtained from International Union for the Conservation of Nature (IUCN) website (https://www.iucnredlist.org/). The boundary was obtained from Natural Earth (http://www.naturalearthdata.com/), a schematic line illustrating the relative position of each country and should not be re-used or misinterpreted for any political reason. The data used for this figure is under CC BY license, and permission for its use has been obtained from the IUCN.

The LCP analysis revealed eight potential transboundary paths (Fig 5) in the research area. The eight identified livestock transboundary paths were: A. Mandi (India)-Ali region (Tibet, China); B. Almora (India)-Ali region (Tibet, China); C. Khalanga (Nepal)-Ali region (Tibet, China); D. Chukha (Bhutan)-Lhasa (Tibet, China); E. Chandpur (Bangladesh) -India-Burma-Dali (Yunnan, China); F. Mahakali (Nepal)—Uttarakhand/Uttar Pradesh (India); G. Seti (Nepal)—Uttar Pradesh (India); H. Lumbini (Nepal)—Uttar Pradesh/ Madhya Pradesh (India).

**Fig 6:**
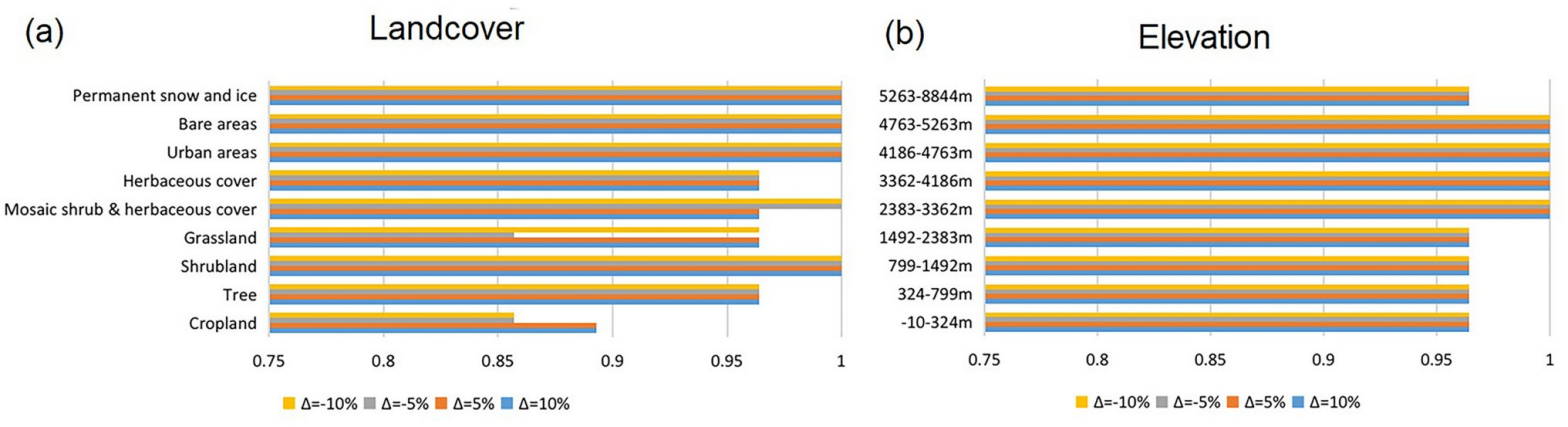
Bar chart of land cover (a) and elevation (b) cost value sensitivity analysis. Spearman rank correlation coefficient between the ranking obtained with the original cost value and the rankings obtained with small variation Δ applied on the original values ranging between -10% and +10% when applicable.

The cost value of each classification is changed by 5% (incremental percent change) within the range of −10% to +10% (range percent change). Thus, the sensitivity analysis consisted of 72 model iterations. The results are visually represented by comparing the iterative models with the original cost value model through the Spearman rank correlation coefficient, as shown in Fig 6. Fig 6(a) and 6(b) indicate that the cost value of elevation has little effect on ranking. In contrast, the sensitivity of the cost value of land cover is only slightly higher than the former (see S3 and S4 Tables for more details). Sensitivity analysis underlined the significant stability of the rankings with respect to the variation in the cost value for the land cover and elevation perspectives.

## Discussion

### PPR risk and variable analysis

Human population density is the most important predictive variable in the low-elevation model, with a contribution of 58.2%. Indeed, the low-elevation areas in this study are mainly distributed in the Indus plains, which are heavily populated. Although animal husbandry is the major source of income in this area [82], considering the large local population of the herds, both the variables of sheep and goat density were excluded by the MaxEnt model due to their low contribution rate (0–0.1). However, this does not indicate that host density has nothing to do with PPR risk. It may also be due to the correlation between human and host density that leads to the deletion of collinear variables in the model, which needs further investigation. In contrast, human population densities were considered as the third highest prediction variable in the high-elevation model. Nonetheless, the response curves of the human population density for both models displayed a similar trend, with a rapid increase in PPR incidence as the population density increases, followed by a plateau. Therefore, despite the variations in the contribution of human population density in both models, its close correlation with the incidence of PPR cannot be overlooked.

The temperature has also been suggested to play an important role in transmitting and spreading infectious diseases [83]. In this study, the mean temperature in April had the highest contribution rate in the high-elevation model. Small ruminants in the Tibetan plateau are mainly raised by transhumance, and summer pastures in high-elevation areas are commonly used during the warm season (April to May) [84]. Transhumance and migration of wildlife have intensified the direct and indirect contact between wildlife and livestock, which might increase the risk of PPRV transmission. In contrast, domestic small ruminants are kept in farms in low-elevation areas. The effect of seasonal temperatures was minimized accordingly. However, the response curves remind us that the appropriate temperature in summer (Min T Aug.: 15–30°C) and winter (Min T Nov.: 0–20°C) can increase the risk of PPR. These alerted us to pay additional attention to seasonality in preventing PPR, especially the risks of seasonal pasture transfer in transhumance areas.

Many landform variables related to gathering contributed to the prediction of the PPR risk in our models. According to the response curves, habitats with deciduous broad-leaved forests, urban areas, or shrublands had the highest probability of PPR. Both the deciduous broad-leaved forests and shrublands could provide food and shelter for small ruminants. In addition, they are mainly distributed in the temperate zone, which is consistent with the optimum temperature shown by the climate variables. The insignificant importance of the slope angle to our models can be explained by the good climbing skill of small ruminants, i.e., the slope hardly restricted their distribution [85]. For this reason, slope angles were not included in our LCP model.

The variations in the distances to the river contributed significantly in the high elevation model (8.9%) than in the low elevation model (1.8%). From the high elevation areas response curve, it could be understood that a farther distance from the river decreases the risk. This is different from that in the low elevation areas. The high-risk areas distributed along the rivers in Tibet (Fig 4) can be explained by the prevalence of a cluster of wildlife around the water holes, which would increase contact and spread of PPRV. The accessibility of water resources and the lush vegetation in plain areas dispense the need for rivers.

Our model shows that the mixed crop-livestock farming system has a very high PPR risk in areas close to the Himalayas, and human influence (population) is the main variable in such cases. Most ruminants in mixed crop-livestock farming systems are found in rural areas and have frequent contact with farmers due to production demand. Therefore, the risk of PPR being dominated by the human impact is expected. For grazing systems, high-risk areas are only scattered around the river valley, and the natural environment (temperature) is the dominant variable. Transhumance became the link between temperature, ruminants, and PPRV. The communication of risk between the two livestock systems and two different landscapes may play a potential role in driving PPR transboundary transmission.

### The impact of wild and domestic hosts on PPR

The host populations are important for PPR maintenance, bridges, and transmission. Because of the complex migration of wild susceptible hosts, obtaining high-quality data for model construction is not easy. However, population profiles (see the second paragraph of the study area section for more detail) can still help us analyze its impact on PPR. In Fig 5, we observe that bharal is distributed in the Tibetan plateau, providing sufficient bridge hosts. Himalayan goral occupies the Himalayas with a high population density. In contrast, the populations of blackbuck and markhor are relatively small. Moreover, the other seven paths, except path E, are within the territory range of wild small ruminants, which might become bridge hosts for PPR transboundary transmission.

### LCP

The LCP analysis returned eight transboundary paths between India, Bhutan, Bangladesh, Nepal, and China. Next, we describe the two-way communication of PPR risk from outside China to inner China. One end of Path A connects to Mandi city in northwest India, which is known to have a prosperous livestock industry with large populations of small ruminants but poor animal health and veterinary services [86]. The predicted risk of PPR is extremely high in this area. Path A further extends southwest to the Himalayas and passes through the middle section of the Sino-Indian border into China. While the elevation along path A is generally high with a peak of 5733 m, many wild ruminants (viz. bharal and Himalayan goral) can cross such rugged terrain. At the other end of path A is the vast alpine pasture area of the Tibetan Plateau, where nomadic domestic small ruminants are widely distributed, which provides a sufficient host for PPRV. Path B is like A, from northwestern India to Tibet, but its length is shorter, and it might be the fastest path contributing to the spread of PPR across borders. Path C extends from midwest Nepal, where PPR frequently occurs, to Tibet. In this path, the porous border and unrestricted animal movement within the country during festive seasons (August to October) may also aid in spreading the disease [87]. Path D extends from Chukha (Bhutan) along the river valley to Lhasa (Tibet), with wild small ruminants distributed along the way. At one end of path E is Chandpur (Bangladesh) that follows the Jamuna River and the Brahmaputra rivers to Parshuram Kund (India), and then crosses the Burmese section of the Arakan Mountains to Dali (Yunnan, China). This path is also mainly distributed along the river valley, and the bushes on both sides of the valley make it easier for the animals to cross. Both paths F and G start from the edge of the Himalayas (within Nepal) and reach the Ganges plain. Path H crosses the Ganges plain and extends to the Indian plain in central India, and the upper two paths are in low elevation areas, which do not offer any obstruction to the movement of small ruminants.

The constructed LCP model involved the main variables that affect the movement of ruminants. The complicated secondary variables (such as hunting and natural enemies) were not included because they could not be measured. At the same time, the merits of the LCP model do exist, especially for a large geographical scale prediction. The cost value commonly depends on the literature and the researchers. It is worth noting that identifying the animal corridors is not easy. LCP is still an effective and universal quantitative method to solve this problem [88]. Mutual verification between the model and reference [89] confirmed that the sensitivity of the LCP model is resistant to slight changes in the values of variables. We put forward a set of methods for countries with data limitations and regions too vast and/or too difficult to access to provide a quick risk assessment.

## Supporting information

S1 Table Elevation classification and cost value. (DOCX)

S2 Table Land cover type and cost value. (DOCX)

S3 Table Land cover cost value sensitivity analysis. (DOCX)

S4 Table Elevation cost value sensitivity analysis. (DOCX)
