# Supplementary material for: A predictive analysis on the risk of peste des petits ruminants in livestock in the Trans-Himalayan region and validation of its transboundary transmission paths
Source: PLoS One. 2021 Sep 10;16(9):e0257094. doi: 10.1371/journal.pone.0257094 (PMC8432769; doi:10.1371/journal.pone.0257094)
Supplement: S2 Table — (DOCX) [file pone.0257094.s002.docx]

**S2 Table. Land cover type and cost value**

| Landcover type | Cost value |
| --- | --- |
| Herbaceous cover | 1 |
| Mosaic shrub & herbaceous cover | 2 |
| Shrubland | 3 |
| Grassland | 4 |
| Tree | 5 |
| Cropland | 6 |
| Bare areas | 7 |
| Permanent snow and ice | 8 |
| Urban areas | 9 |
| Water bodies | Restricted |
