# Supplementary material for: A predictive analysis on the risk of peste des petits ruminants in livestock in the Trans-Himalayan region and validation of its transboundary transmission paths
Source: PLoS One. 2021 Sep 10;16(9):e0257094. doi: 10.1371/journal.pone.0257094 (PMC8432769; doi:10.1371/journal.pone.0257094)
Supplement: S3 Table — (DOCX) [file pone.0257094.s003.docx]

**S3 Table. Land cover cost value sensitivity analysis.**

| Land cover type | Δ=10% | Δ=5% | Δ=-5% | Δ=-10% |
| --- | --- | --- | --- | --- |
| Cropland | 0.893 | 0.893 | 0.857 | 0.857 |
| Tree | 0.964 | 0.964 | 0.964 | 0.964 |
| Shrubland | 1 | 1 | 1 | 1 |
| Grassland | 0.964 | 0.964 | 0.857 | 0.964 |
| Mosaic shrub & herbaceous cover | 0.964 | 0.964 | 1 | 1 |
| Herbaceous cover | 0.964 | 0.964 | 0.964 | 0.964 |
| Urban areas | 1 | 1 | 1 | 1 |
| Bare areas | 1 | 1 | 1 | 1 |
| Permanent snow and ice | 1 | 1 | 1 | 1 |
