# Supplementary material for: A predictive analysis on the risk of peste des petits ruminants in livestock in the Trans-Himalayan region and validation of its transboundary transmission paths
Source: PLoS One. 2021 Sep 10;16(9):e0257094. doi: 10.1371/journal.pone.0257094 (PMC8432769; doi:10.1371/journal.pone.0257094)
Supplement: S4 Table — (DOCX) [file pone.0257094.s004.docx]

**S4 Table. Elevation cost value sensitivity analysis**

| Elevation classification | Δ=10% | Δ=5% | Δ=-5% | Δ=-10% |
| --- | --- | --- | --- | --- |
| -10-308m | 0.964 | 0.964 | 0.964 | 0.964 |
| 308-746m | 0.964 | 0.964 | 0.964 | 0.964 |
| 746-1383m | 0.964 | 0.964 | 0.964 | 0.964 |
| 1383-2211m | 0.964 | 0.964 | 0.964 | 0.964 |
| 2211-3084m | 1 | 1 | 1 | 1 |
| 3084-3847m | 1 | 1 | 1 | 1 |
| 3847-4440m | 1 | 1 | 1 | 1 |
| 4440-4873m | 1 | 1 | 1 | 1 |
| 4873-5332m | 1 | 1 | 1 | 1 |
| 5332-8844m | 0.964 | 0.964 | 0.964 | 0.964 |
